# Health facility determinants and trends of ICD-10 outpatient psychiatric consultations across Sofala, Mozambique: time-series analyses from 2012 to 2014

**DOI:** 10.1186/s12888-015-0609-4

**Published:** 2015-09-23

**Authors:** Bradley H. Wagenaar, Vasco Cumbe, Manuela Raunig-Berhó, Deepa Rao, Manuel Napúa, James P. Hughes, Kenneth Sherr

**Affiliations:** Department of Epidemiology, University of Washington School of Public Health, 1959 NE Pacific Street, Seattle, WA 98195 USA; Health Alliance International, Seattle, Washington USA; Department of Mental Health, Ministry of Health, Sofala Provincial Health Directorate, Beira, Mozambique; Department of Psychiatry, Beira Central Hospital, Beira, Mozambique; Department of Global Health, University of Washington, Seattle, WA USA; Department of Psychiatry and Behavioral Sciences, University of Washington, Seattle, WA USA; Beira Operations Research Center, Ministry of Health, Beira, Mozambique; Department of Biostatistics, University of Washington, Seattle, WA USA

**Keywords:** Mental health services, Outpatient psychiatry, Health facility determinants, Time-trends, Gender differences, Mozambique

## Abstract

**Background:**

Few peer-reviewed publications have taken a longitudinal or systems approach to mental healthcare (MH) utilization in low- and middle-income countries. We analyzed: (1) outpatient ICD-10 diagnoses over time and by gender; and (2) health facility determinants of MH service utilization.

**Methods:**

We reviewed a census of 15,856 outpatient psychiatric consultations conducted at Ministry clinics in Sofala province, Mozambique from January 2012-June 2014. Generalized estimating equations were used to model facility determinants of ICD-10 diagnoses.

**Results:**

Across the period, 48.9 % of consults were for epilepsy, 22.4 % for schizophrenia/delusional disorders, and 8.8 % for neurotic/stress-related disorders. The proportion of schizophrenia/delusional disorders has decreased over time (32 % in 2012; 13 % in 2014, *p* = 0.003), in favor of greater diversity of diagnoses. Epilepsy has increased significantly in absolute and proportional terms. Women are more likely to present for neurotic/stress-related conditions (12.8 % of consults for women, 5.7 % for men, *p* < 0.001), while men are more likely to present for substance use (1.9 % for women, 6.4 % for men, *p* < 0.001). Clinics with more psychiatric technicians have a 2.1-fold (CI: 1.2, 3.6) increased rate of schizophrenia/delusional disorder diagnoses. Rural clinics saw a higher proportion of epilepsy cases and a lower proportion of organic, substance use, schizophrenia, and mood disorder cases.

**Discussion and Conclusions:**

Outpatient MH service provision is increasing in Mozambique, although currently focuses on epilepsy and schizophrenia/delusional disorders. Mid-level psychiatric providers appear to be associated with a higher proportion of schizophrenia/delusional disorder diagnoses. Due to diagnostic or utilization differences, rural clinics may be missing important cases of organic, substance use, schizophrenia, and mood disorders. Models and decision-support tools for mental healthcare integration with primary care practice are needed in Mozambique to allow further scale-up of mental health services.

## Background

With the advent of the first global burden of disease studies in the early 1990s, and the development of standardized metrics of “burden” in disability-adjusted life years (DALYs) or years lived with disability (YLDs), mental, neurological, and substance-use disorders began to be understood as a leading, if not *the* leading cause of disability worldwide [1]. This culminated in the 2001 World Health Report on global mental health, with an urgent call to increase resource allocation and service provision for mental health in primary care settings globally [2]. Over 7 years ago there appeared to be international agreement that there can be “no health without mental health” [3], yet the most updated World Health Organization (WHO) mental health atlas estimates that the average percent of health budgets allocated to mental health continues to stand at a shockingly low 0.62 % in countries in the WHO African region [4, 5].

Across Sub-Saharan Africa as a whole there are few peer-reviewed studies detailing current systems profiles for mental healthcare provision, such as: 1) *who* is currently being served and *who* may be missed?; 2) *what* disorders are people presenting with and *what* are they being treated with?; 3) *where* are services currently located and *where* are the highest burden of patients located?; 4) *how* are these systems currently organized (human resources, training programs, supply chains, financing, etc.); and *how* can we target future systems improvement approaches? These foundational systems-level analyses should help inform community studies of incidence/prevalence and mental health risk factors to determine priority conditions and targeted expansion of mental healthcare systems in low-and middle-income countries (LMICs).

Mozambique, in particular, was recently estimated to have the 7th highest suicide rate in the world [6], yet currently only 7.2 % of public health facilities offer any services for mental health, and more than half of all districts in the country contain no facility offering mental health services [7]. Given this low coverage, it is estimated that only 0.29 % of the population has reliable access to basic mental health services [8]. While there has been much international discussion around implementing task-sharing approaches to scale-up care and treatment for common mental disorders in primary care settings [9–12], what literature is available has mostly focused on testing the feasibility and efficacy of task-sharing approaches under controlled settings [13–16]. The WHO *Mental Health Gap Action Programme* (mhGAP) aims to promote the scaling-up of prevention and treatment for mental disorders in LMICs using task-sharing approaches, but the programme remains to be adapted or rolled-out in many countries, including Mozambique. The widely used mhGAP training materials [17] are not currently available in Portuguese – a barrier to adoption for most mid-level mental health providers in Mozambique.

Over a decade and a half ago the Mozambican Ministry of Health rolled-out a task-sharing approach through the development of a two-year psychiatric technician training program for graduates of at least the 10th grade. These technicians currently provide the vast majority of psychiatric services nationwide, diagnosing conditions and prescribing psychotropic medications. However, there have not been any systematic studies regarding the effectiveness of care provided by this growing and essential cadre of health workers in Mozambique.

Limited available mental health system data from Mozambique indicate that epilepsy is the leading diagnosis in outpatient consultations (53 % of consultations), followed by child mental disorders (15 %), and schizophrenia (14 %) [7]. Regarding inpatient services, in one sample, 47 % were diagnosed with schizophrenia, 31 % with epilepsy or other organic disorders, and 18 % with substance use. In Zambia, acute/transient psychotic states are the most common outpatient diagnosis, followed by schizophrenia, substance use, and dementia [18]. In Nigeria, 10.4 % of patients attending a general outpatient clinic had a mental disorder as classified by the International Classification of Diseases and Related Health Problems, Tenth edition (ICD-10) [19], with depression being the most common at 4 %, followed by generalized anxiety disorder (3 %) [20].

The objective of the present study is to fill a gap in the peer-reviewed literature, aiming to: 1) assess diagnostic patterns in mental healthcare utilization; 2) analyze trends in utilization over the past two years; and 3) identify potential health facility determinants of mental healthcare diagnosis and utilization, all across a census of health facilities providing mental health services in Sofala Province, Mozambique. We hope these data can inform the scale-up of mental healthcare provision in Mozambique, as well as other similar LMIC settings.

## Methods

### Study setting

Following independence from Portugual in 1975, Mozambique began transitioning from mental healthcare mostly provided in asylums to a more decentralized community-based mental healthcare system. The first national seminar on mental health took place in 1984, highlighting the importance of: 1) decentralizing treatment facilities; 2) gathering data on the epidemiologic distribution of disorders in the country; 3) investing in human resources for mental health; 4) raising awareness about mental illness in the country; and 5) building multi-sectoral collaborations [7]. The national mental health program was formally adopted in 1990, which created the psychiatric technician training program, one of the first examples of task-sharing programs for mental health. The first cadre of 34 psychiatric technicians began service in 1996, with refresher courses rolled-out in 2005 [7].

From 1990 to 1996 the mental health care system expanded from 6 to 24 care centers nationally. Yet, the availability of services was still severely limited due to the lack of human resources, namely only one Mozambican, and two foreign psychiatrists [7]. Following the graduation of the first cadre of psychiatric technicians, outpatient psychiatric care evolved to all 11 provinces, primarily at large central or district-level referral hospitals. An additional 31 psychiatric technicians graduated in 2006, and the Ministry of Health has since offered the course every two years, graduating an average of 30 students per class with the goal of expanding psychiatric care to all districts nationally [7]. This goal is close to being met: as of December 2014 there were 241 psychiatric technicians operating across 128 districts nationally [21].

**Fig. 1:**
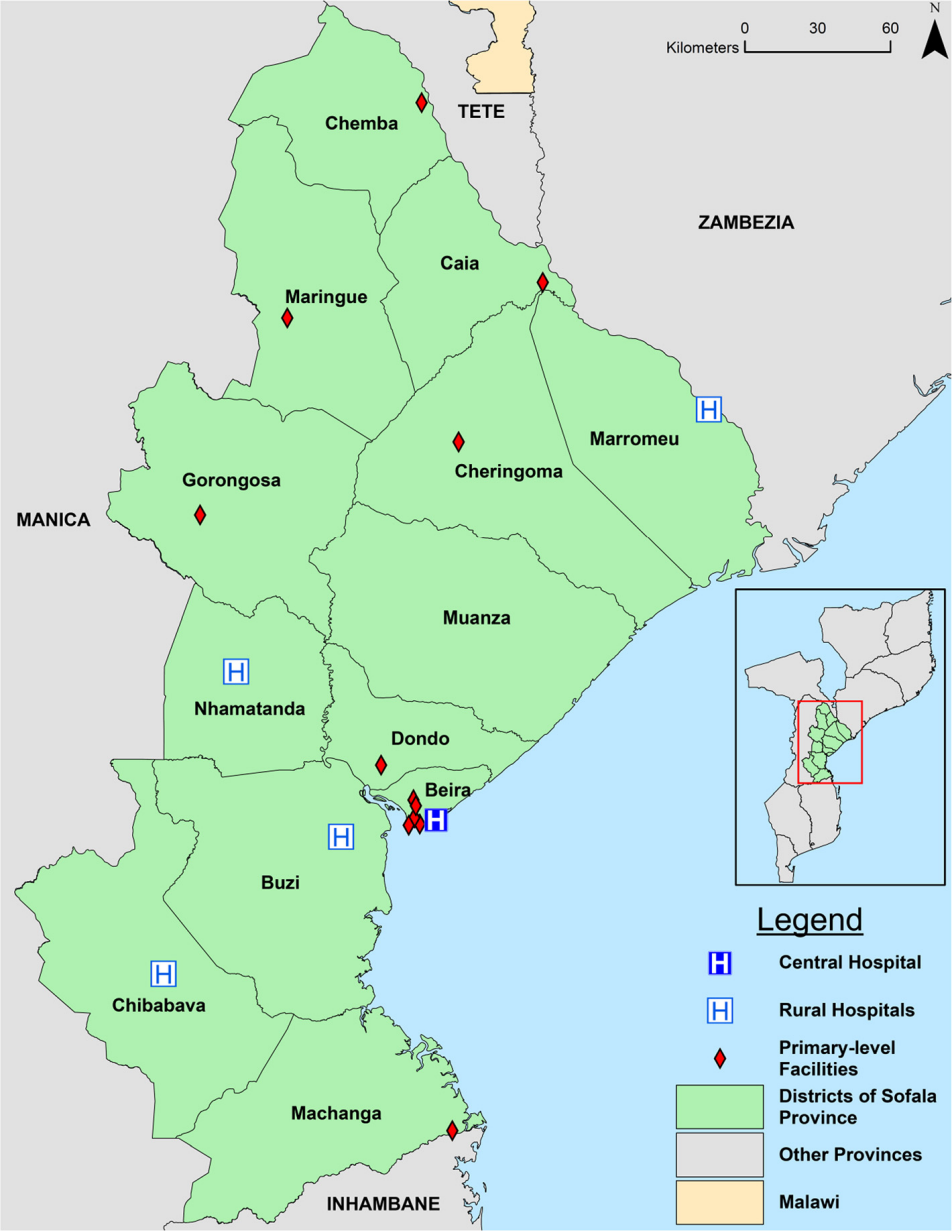
Map of health facilities providing outpatient mental healthcare services in Sofala Province, Mozambique, as of 2014

Currently, Sofala province has at least one facility providing outpatient mental healthcare in 12 of 13 districts. With the exception of Beira City which houses 7 clinics, each district houses one facility with trained mental health professionals conducting routine outpatient consultations (thus, 18 total clinics providing services across the province; see Fig. 1 for a map of mental health facilities). Outside of Beira City, all facilities providing mental healthcare are located at the largest district-level facility (generally a district or rural hospital). Two separate clinics report from the Beira Central Hospital: the general psychiatric service and the child psychiatric service. In the province as a whole, as of the end of 2014, there were 14 psychiatric technicians, 2 adult psychiatrists, 1 child psychiatrist, and 11 clinical psychologists. Mental health diagnoses across all of Mozambique are made using the ICD-10 disease classification system [19].

### Data sources and variables

We abstracted a census of outpatient mental healthcare utilization for all public-sector, Ministry-supported clinics in Sofala province from January 2012 – June 2014. Data are monthly aggregate counts of facility-level utilization broken down by ICD-10 code and gender. Due to changes in reporting, aggregate statistics are available for the province as a whole from January 2012 – June 2014, but facility-level data are only available from October 2012 – June 2014. Data are reported monthly at the district level, with child and adult services at Beira Central Hospital each reporting separately from other clinics in Beira City. This results in facility-level monthly data because all districts except Beira City (5 clinics reporting in Beira City) have only one health facility currently providing mental health services. For this reason, the 5 clinics in Beira City are included in provincial-level aggregate statistics but excluded for facility-level associative analyses explained below.

### Data analyses

Data were analyzed in aggregate to: (1) determine overall trends in mental healthcare utilization from January 2012 – June 2014 by ICD-10 diagnostic category; and (2) to compare utilization figures by gender across this time period. For all aggregate analyses across the 18 operating MH clinics, trends and gender differences were computed for absolute differences in the number of outpatient consultations across time, as well as the proportion of outpatient consultations attributable to each ICD-10 diagnostic category. Prais-Winsten linear regression was used to account for autocorrelation in aggregate utilization figures by ICD-10 grouping while testing for changes across time and for gender differences in ICD-10 absolute or proportional diagnoses.

Facility-level data were used to examine health facility determinants of ICD-10 outpatient psychiatric utilization from October 2012 – June 2014. We chose to use the outcome of the proportion of consultations attributable to each ICD-10 category in order to answer the question of whether patterns of outpatient mental health diagnoses/utilization differ by health facility characteristics. Available health facility characteristics included: number of yearly general outpatient consultations, rural/urban clinic location, level of health facility (primary, secondary, tertiary, quaternary), and mental health staffing (number of psychiatric technicians, psychologists, or psychiatrists). General outpatient (non-mental health) consultation data were abstracted from the national health information system (*Módulo Básico*) and estimates for 2014 were generated by doubling the first 6 months of utilization data. Rural or urban clinic location was determined by Ministry of Health official facility classifications. We abstracted the level of health facility from official Ministry of Health documents and author Cumbe, V. provided mental health staffing numbers.

We used negative binomial generalized estimating equations to model the fully-adjusted effect of each predictor simultaneously on the count of individual ICD-10 classification groups. An offset term was used equal to the natural log of the denominator of total ICD-10 consults. An exchangeable working correlation matrix and robust standard errors were used, with data clustered at the facility level. All analyses used Stata 13 and an alpha value of 0.05. Due to small numbers of diagnoses leading to instability of model estimates and difficulty in model convergence, models were only built for ICD-10 categories achiving >15 monthly consults, and mental health staffing variables were excluded for all ICD-10 diagnostic categories, save schizophrenia/delusional disorders and epilepsy.

### Ethical approval

This study was approved by the institutional review boards at the University of Washington and the Mozambican National Institute of Health. Permission to use these data were obtained through the Mozambican National Institute of Health and by author Cumbe, V. in his role as the head of the Department of Mental Health for Sofala Province.

## Results

Clinics providing mental health services had an average of 78,418 general outpatient (non-mental health) consultations per clinic from Jan 2012 – June 2014 (see Table 1). Over 55 % of clinics were in rural locations (10 of 18 clinics) and the majority (67 %) were primary level facilities (Table 1). Of those clinics with complete staffing information (13 of 18), 85 % (11 of 13) had no psychiatrists, 69 % (9 of 13) had no psychologists, and 77 % (10 of 13) had one psychiatric technician. Excluding those clinics missing mental health staffing information, 57 % (7,462 of 13,093) of outpatient consultations were conducted at a clinic with one psychiatric technician, no psychologists, and no psychiatrists.

**Table 1:** Demographic characteristics of 14 health facilities providing outpatient mental healthcare for the period of January 2012-June 2014, Sofala Province, Mozambique

Characteristic	Number of clinics, n (%) unless noted	Number outpatient mental health consultations n (%)
*Total*	*18 (100)*	*15,856* ^a^ *(100)*
Yearly general outpatient consults 2012–2014, Mean (SD)	78,418 (45,782)	n/a
Rural clinic location	10 (55.6)	8,869 (55.9)
*Type of health facility*		
Central Hospital	2 (11.1)	4,069 (25.7)
Urban Health Center – Type A	6 (33.3)	2,918 (18.4)
Rural Hospital	4 (22.2)	5,686 (35.9)
Rural Health Center – Type 2	0 (0)	0 (0)
Rural Health Center – Type 1	6 (33.3)	3,183 (20.1)
*Level of health facility* ^a^		
Primary	12 (66.7)	6,101 (38.5)
Secondary	4 (22.2)	5,686 (35.9)
Tertiary	0 (0)	0 (0)
Quaternary	2 (11.1)	4,069 (25.7)
*Mental health staffing in 2014* ^b^		
Psychiatrists		
0	11 (61.1)	9,024 (56.9)
1	1 (5.6)	873 (5.5)
2	1 (5.6)	3,196 (20.2)
Missing (Beira City)	5 (27.8)	2,763 (17.4)
Psychologists		
0	9 (50.0)	7,882 (49.7)
1	2 (11.1)	1,142 (7.2)
2	0 (0)	2,746 (17.3)
3	2 (11.1)	1,323 (8.3)
Missing (Beira City)	5 (27.8)	2,763 (17.4)
Psychiatric technicians		
0	2 (11.1)	1,293 (8.2)
1	10 (55.6)	8,604 (54.3)
2	1 (5.6)	3,196 (20.2)
Missing (Beira City)	5 (27.8)	2,763 (17.4)

^a^Primary-level facilities conduct outpatient, prenatal, well-child, and maternity services with some larger facilities having x-ray capabilities, minor emergency care, and inpatient beds. Secondary-level facilities provide some specialized care including advanced laboratory, radiology capabilities, blood banks, and major surgical wards. Quaternary-level facilities have a large staff of healthcare workers including numerous specialties such as neurology, cardiology, neuro-surgery, oncology, psychiatry, etc

^b^Beira city is missing because data are reported in aggregate for all facilities

### Aggregate trends over time across all public clinics providing mental health services

**Fig. 2:**
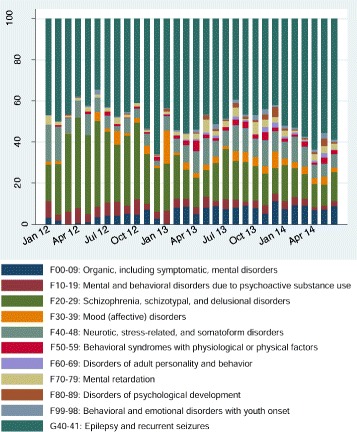
Proportion of outpatient mental health consultations by ICD-10 diagnosis code in Sofala Province, Mozambique from January 2012 to June 2014

Across Sofala Province, mental health services conducted 6,629 outpatient consultations in 2012, 8,522 in 2013, and 5,858 through June of 2014, representing a significant increase in total consultations (from 552 per month in 2012 to 976 in 2014, p < 0.001); (Table 2). Absolute utilization numbers increased significantly for most ICD-10 sub-groups, except no significant change in utilization for mental and behavioral disorders due to substance use and a significant decrease for schizophrenia, schizotypal, and delusional disorders (177.4 consults per month in 2012 to 125.8 in 2014, p = 0.045). The proportion of consults increased significantly for many ICD-10 categories, save a proportional decrease for mental and behavioral disorders due to substance use, a large proportional decrease in schizophrenia, schizotypal, and delusional disorders, and no change for mood disorders, neurotic and stress-related disorders, and mental retardation (See Table 2 and Fig. 2).

**Table 2:** Change in the distribution of outpatient consultations using ICD-10 for mental disorders in Sofala, Mozambique, January 2012 – June 2014

ICD-10 Diagnostic Category	Change in # of monthly consults	*p*-value	Mean # monthly consults	Change in proportion of consults	*p*-value	Mean proportion of consults (SD)
	β (95 % CI)		(SD)	β (95 % CI)		
*Total*	n/a		700.3 (195.9)	n/a		100
Jan – Dec 2012	0 (reference)		552.4 (125.2)	n/a		100
Jan – Dec 2013	153.9 (72.7, 235.2)	<0.001	710.2 (96.4)	n/a		100
Jan – June 2014	426.0 (326.1, 525.9)	<0.001	976.3 (153.0)	n/a		100
F00-F09: Organic, including symptomatic, mental disorders			43.8 (28.7)			5.8 (3.1)
Jan – Dec 2012	0 (reference)		17.8 (11.7)	0 (reference)		3.2 (2.1)
Jan – Dec 2013	34.6 (20.7, 48.4)	<0.001	52.3 (20.7)	4.0 (1.9, 6.1)	<0.001	7.3 (2.8)
Jan – June 2014	61.0 (44.0, 78.0)	<0.001	78.7 (16.6)	4.7 (2.1, 7.2)	<0.001	8.1 (1.1)
F10-F19: Mental and behavioral disorders due to psychoactive substance use			28.9 (9.3)			4.4 (1.7)
Jan – Dec 2012	0 (reference)		29.7 (11.6)	0 (reference)		5.4 (1.8)
Jan – Dec 2013	−1.8 (−8.2, 4.6)	0.573	28.4 (7.1)	−1.4 (−2.6, −0.14)	0.003	4.1 (1.2)
Jan – June 2014	−1.7 (−9.5, 6.2)	0.665	28.3 (9.3)	−2.5 (−3.9, −1.0)	<0.001	2.9 (0.94)
F20-F29: Schizophrenia, schizotypal, and delusional disorders			145.9 (50.6)			22.4 (10.0)
Jan – Dec 2012	0 (reference)		177.4 (61.7)	0 (reference)		31.9 (8.6)
Jan – Dec 2013	−51.6 (−91.3, −11.9)	0.013	124.5 (31.2)	−11.4 (−18.8, −3.9)	0.004	17.5 (3.9)
Jan – June 2014	−49.7 (−98.3, −1.1)	0.045	125.8 (19.4)	−14.5 (−23.7, −5.3)	0.003	13.1 (2.7)
F30-F39: Mood (affective) disorders			24.3 (21.0)			3.3 (3.1)
Jan – Dec 2012	0 (reference)		12 (14.3)	0 (reference)		2.0 (2.1)
Jan – Dec 2013	19.2 (5.7, 32.8)	0.007	33.3 (25.2)	2.4 (0.36, 4.5)	0.023	4.8 (4.1)
Jan – June 2014	19.0 (2.4, 35.7)	0.026	31.0 (10.3)	1.0 (−1.5, 3.6)	0.400	3.1 (0.60)
F40-F48: Neurotic, stress-related, and somatoform disorders			61.6 (20.0)			8.8 (3.5)
Jan – Dec 2012	0 (reference)		44.5 (31.4)	0 (reference)		8.1 (4.9)
Jan – Dec 2013	24.7 (−1.6, 51.0)	0.065	69.2 (21.3)	1.6 (−2.5, 5.7)	0.438	9.7 (2.2)
Jan – June 2014	34.1 (1.9, 66.3)	0.039	80.5 (21.2)	−0.20 (−5.3, 4.9)	0.935	8.4 (2.4)
F50-F59: Behavioral syndromes associated with physiological disturbances and physical factors			11.8 (9.9)			1.6 (1.2)
Jan – Dec 2012	0 (reference)		4.0 (3.2)	0 (reference)		0.72 (0.59)
Jan – Dec 2013	13.1 (8.1, 18.2)	<0.001	16.8 (9.6)	1.6 (1.0, 2.3)	<0.001	2.3 (1.2)
Jan – June 2014	13.7 (7.5, 20.0)	<0.001	17.5 (9.8)	1.0 (0.22, 1.8)	0.015	1.7 (0.75)
F60-F69: Disorders of adult personality and behavior			4.8 (6.9)			0.58 (0.78)
Jan – Dec 2012	0 (reference)		0 (0)	0 (reference)		0 (0)
Jan – Dec 2013	7.1 (2.7, 11.5)	0.003	7.1 (6.8)	0.94 (0.41, 1.5)	0.001	0.94 (0.83)
Jan – June 2014	10.2 (4.8, 15.7)	0.001	10 (9.4)	1.0 (0.37, 1.7)	0.003	1.0 (0.79)
F70-F79: Mental retardation			17.3 (11.8)			2.4 (1.5)
Jan – Dec 2012	0 (reference)		10.2 (6.3)	0 (reference)		1.8 (1.1)
Jan – Dec 2013	11.9 (3.2, 20.7)	0.009	22.1 (12.7)	1.3 (0.20, 2.4)	0.022	3.1 (1.7)
Jan – June 2014	12.0 (1.3, 22.7)	0.029	22.2 (12.4)	0.46 (−0.88, 1.8)	0.486	2.3 (1.2)
F80-F89: Disorders of psychological development			7.2 (9.7)			0.91 (1.1)
Jan – Dec 2012	0 (reference)		0.83 (1.2)	0 (reference)		0.16 (0.23)
Jan – Dec 2013	10.2 (2.8, 17.7)	0.009	10.9 (9.9)	1.4 (0.47, 2.3)	0.004	1.5 (1.3)
Jan – June 2014	11.2 (2.1, 20.4)	0.018	12.5 (12.7)	0.98 (−0.12, 2.1)	0.079	1.2 (1.2)
F90-F98: Behavioral and emotional disorders with onset usually occurring in childhood and adolescence			7.5 (7.5)			0.95 (0.84)
Jan – Dec 2012	0 (reference)		2.7 (3.6)	0 (reference)		0.48 (0.62)
Jan – Dec 2013	6.3 (1.2, 11.4)	0.018	8.9 (7.3)	0.70 (0.06, 1.3)	0.033	1.2 (0.89)
Jan – June 2014	11.7 (5.4, 17.9)	0.001	14.3 (7.9)	0.97 (0.20, 1.8)	0.016	1.5 (0.70)
G40-41: Epilepsy and recurrent seizures			347.1 (130.6)			48.9 (7.4)
Jan – Dec 2012	0 (reference)		253.3 (65.9)	0 (reference)		46.2 (8.1)
Jan – Dec 2013	84.4 (34.4, 134.4)	0.002	336.8 (45.1)	−0.83 (−7.8, 6.1)	0.809	47.7 (5.2)
Jan – June 2014	304.1 (242.7, 365.4)	<0.001	555.5 (107.1)	9.4 (0.89, 18.0)	0.032	56.7 (4.3)

### Gender comparison for distribution of ICD-10 outpatient consultations

In absolute and proportional terms, significantly more males presented at outpatient psychiatric visits for mental and behavioral disorders due to substance use, with these conditions comprising 6.4 % of all consultations for men, but only 1.9 % for women (p < 0.001); (Table 3). This trend was reversed when considering neurotic, stress-related, and somatoform disorders, which comprised 12.8 % of all consultations for women and only 5.7 % for men (p < 0.001).

**Table 3:** Gender comparison of the distribution of outpatient consultations using ICD-10 for mental disorders in Sofala, Mozambique, January 2012 – June 2014

ICD-10 Diagnostic Category	Absolute gender difference	*p*-value	Mean # monthly consults	Proportion gender difference	*p*-value	Mean proportion of consults (SD)
	β (95 % CI)		(SD)	β (95 % CI)		
*Total Male*	19.8 (−73.4, 113.0)	0.671	362.8 (103.8)	n/a		100
*Total Female*	0 (reference)		344.7 (102.3)	n/a		100
F00-F09: Organic, including symptomatic, mental disorders						
Male	−0.12 (−16.6, 16.4)	0.988	22.3 (14.4)	−0.19 (−1.5, 1.1)	0.780	5.9 (3.3)
Female	0 (reference)		23.1 (16.9)	0 (reference)		6.0 (3.6)
F10-F19: Mental and behavioral disorders due to psychoactive substance use						
Male	15.3 (12.7, 17.8)	<0.001	21.7 (7.2)	4.5 (3.5, 5.5)	<0.001	6.4 (2.8)
Female	0 (reference)		6.4 (3.2)	0 (reference)		1.9 (0.9)
F20-F29: Schizophrenia, schizotypal, and delusional disorders						
Male	9.3 (−8.1, 26.8)	0.288	74.4 (27.0)	1.9 (−3.9, 7.6)	0.523	22.1 (10.3)
Female	0 (reference)		65.4 (24.2)	0 (reference)		20.5 (9.9)
F30-F39: Mood (affective) disorders						
Male	−4.2 (−10.6, 2.2)	0.192	10.2 (11.8)	−1.2 (−2.8, 0.30)	0.113	2.7 (3.6)
Female	0 (reference)		14.4 (10.5)	0 (reference)		4.0 (3.0)
F40-F48: Neurotic, stress-related, and somatoform disorders						
Male	−22.3 (−33.9, −10.7)	<0.001	21.1 (12.4)	−7.2 (−10.1, −4.4)	<0.001	5.7 (2.9)
Female	0 (reference)		43.7 (17.9)	0 (reference)		12.8 (5.0)
F50-F59: Behavioral syndromes associated with physiological disturbances and physical factors						
Male	1.9 (−1.7, 5.6)	0.290	7.2 (6.2)	0.47 (−0.20, 1.1)	0.163	1.9 (1.4)
Female	0 (reference)		5.2 (4.4)	0 (reference)		1.4 (1.1)
F60-F69: Disorders of adult personality and behavior						
Male	−0.48 (−3.1, 2.2)	0.718	2.4 (3.3)	−0.12 (−0.48, 0.23)	0.492	0.59 (0.76)
Female	0 (reference)		2.9 (4.0)	0 (reference)		0.71 (0.92)
F70-F79: Mental retardation						
Male	1.6 (−2.7, 5.9)	0.458	9.7 (6.7)	0.34 (−0.58, 1.3)	0.463	2.6 (1.7)
Female	0 (reference)		8.0 (6.1)	0 (reference)		2.3 (1.5)
F80-F89: Disorders of psychological development						
Male	−0.26 (−3.9, 3.4)	0.887	3.8 (5.1)	−0.13 (−0.68, 0.41)	0.631	0.93 (1.2)
Female	0 (reference)		4.1 (5.2)	0 (reference)		1.1 (1.2)
F90-F98: Behavioral and emotional disorders with onset usually occurring in childhood and adolescence						
Male	1.5 (−1.4, 4.4)	0.306	4.8 (4.3)	0.39 (−0.04, 0.83)	0.076	1.2 (1.0)
Female	0 (reference)		3.3 (4.0)	0 (reference)		0.81 (0.86)
G40-41: Epilepsy and recurrent seizures						
Male	17.5 (−64.1, 99.0)	0.669	185.2 (76.5)	1.6 (−3.9, 7.1)	0.564	50.0 (8.7)
Female	0 (reference)		168.3 (61.1)	0 (reference)		48.4 (7.2)

### Health system determinants of the proportional distribution of ICD-10 outpatient consultations

Controlling for other available health facility determinants, larger clinics (those with more general outpatient consultations) had a 10 % (CI: 18 %, 3 %) lower rate of diagnoses for schizophrenia, schizotypal, and delusional disorders per 10,000 increase in general outpatient consultations (Table 4). Compared to urban clinics, those clinics in rural areas had significantly lower rates of consultations for organic disorders (aRR: 0.32; CI: 0.13, 0.75), substance use disorders (aRR: 0.14; CI: 0.06, 0.30), schizophrenia and delusional disorders (aRR: 0.50; CI: 0.40, 0.63), and mood disorders (aRR: 0.22; CI: 0.07, 0.72). By contrast, rural clinics saw a 1.8-fold (CI: 1.2, 2.8) increased rate of consultations for epileptic disorders.

**Table 4:** Health system factors associated with the distribution of outpatient ICD-10 consultations for mental disorders in Sofala, Mozambique, October 2012 – June 2014

Health system factor	F00-F09	*P*-value	F10-F19	*P*-value	F20-F29	*P*-value	F30-F39	*P*-value	F40-F48	*P*-value	F70-F79	*P*-value	G40-41	*P*-value
	aRR^a^ (95 % CI)		aRR^a^ (95 % CI)		aRR^a^ (95 % CI)		aRR^a^ (95 % CI)		aRR^a^ (95 % CI)		aRR^a^ (95 % CI)		aRR^a^ (95 % CI)	
Num. outpatient consultations (per 10,000 increase)	1.06 (0.89, 1.24)	0.52	0.93 (0.84, 1.02)	0.13	0.90 (0.82, 0.97)	0.01	1.2 (0.88, 1.6)	0.26	1.1 (0.93, 1.3)	0.28	1.07 (0.96, 1.2)	0.23	0.98 (0.91, 1.1)	0.56
Rural clinic location	0.32 (0.13, 0.75)	0.009	0.14 (0.06, 0.30)	<0.001	0.50 (0.40, 0.63)	<0.001	0.22 (0.07, 0.72)	0.01	3.5 (0.82, 14.7)	0.09	1.2 (0.45, 3.1)	0.73	1.8 (1.2, 2.8)	0.009
Level of health facility														
Primary	1 (reference)		1 (reference)		1 (reference)		1 (reference)		1 (reference)		1 (reference)		1 (reference)	
Secondary	1.6 (0.46, 5.6)	0.45	0.72 (0.24, 2.2)	0.56	0.95 (0.53, 1.7)	0.85	2.1 (0.72, 6.2)	0.18	0.14 (0.03, 0.69)	0.02	0.23 (0.05, 1.01)	0.05	1.5 (0.93, 2.3)	0.10
Quaternary	1.5 (0.29, 8.0)	0.61	1.36 (0.48, 3.9)	0.57	7.7 (1.3, 44.7)	0.02	0.18 (0.009, 3.3)	0.24	0.59 (0.09, 3.9)	0.58	11.3 (1.8, 71.6)	0.01	0.16 (0.02, 1.3)	0.09
Mental health staffing^b^														
Psychiatrists	Excluded		Excluded		0.71 (0.25, 2.0)	0.52	Excluded		Excluded		Excluded		0.62 (0.27, 1.5)	0.27
Psychologists	Excluded		Excluded		0.68 (0.46, 0.99)	0.05	Excluded		Excluded		Excluded		2.3 (1.2, 4.6)	0.01
Psychiatric technicians	Excluded		Excluded		2.1 (1.2, 3.6)	0.006	Excluded		Excluded		Excluded		0.89 (0.58, 1.4)	0.59

^a^All adjusted rate ratios (aRR) represent fully-adjusted coefficients adjusting for system factors simultaneously in a negative binomial regression model using generalized estimating equations and an exchangeable correlation matrix to control for facility-level correlation

^b^Staffing variables are excluded from F00-F09, F10-F19, F30-F39, F40-F48, and F70-F79 models due to small sample size and instability in estimates if included

The level of health facility was a strong determinant of mental health diagnosis/utilization patterns. Compared to primary-level facilities, quaternary facilities diagnosed schizophrenia and delusional disorders at a 7.7-fold increased rate (CI: 1.3, 44.7) and mental retardation at an 11.3-fold increased rate (CI: 1.8, 71.6). On the other hand, secondary facilities diagnosed neurotic conditions at significantly decreased rates (aRR: 0.14; CI: 0.03, 0.69).

Each additional psychiatric technician was associated with a 2.1-fold (CI: 1.2, 3.6) higher rate of diagnoses for schizophrenia and delusional disorders (Table 4). In addition, each additional psychologist was associated with a 2.3-fold (CI: 1.2, 4.6) increased rate of epilepsy diagnoses.

## Discussion

This study is the first analytical assessment of trends and facility determinants of outpatient mental health diagnoses across a census of public health facilities offering outpatient services in Sofala province, Mozambique. The outpatient mental healthcare system in Mozambique is currently undergoing large-scale change. Our analyses found a rapid increase in the absolute number of outpatient consultations – almost a doubling over the past two years – due to the expansion of care into at least one district-level referral facility in 12 of 13 districts. While this is a major accomplishment, made possible by the increased number of trained psychiatric technicians, the majority of the population still does not have access to routine mental health services. In the absence of epidemiologic measurements on the population prevalence of mental disorders in Mozambique, this mental health treatment gap can be inferred. For example, depression, anxiety, and bipolar disorder are estimated to account for 16.6 % of YLD for those aged 15–49 in Mozambique [22], yet across Sofala Province (population 2 million), there are currently less than 300 yearly consultations for mood disorders.

This expansion of mental health services away from the provincial capital of Beira City and into more rural districts has led to increased utilization and treatment, most notably for epileptic disorders. This is highlighted through a strong association between rural clinic location and a higher rate of patients seen for epilepsy. While epilepsy is not expected to be of higher incidence in rural versus urban areas, the observed relationship is likely due to a combination of epilepsy being relatively easy to diagnose during outpatient consultations, and lower utilization for other less-visible or severe mental health conditions. For instance, we also found a lower rate of consultations in rural areas for substance use disorders. Since a previous population-level study in Mozambique found no rural/urban difference in daily drinking [23], future efforts should potentially target increased screening for alcohol-use disorders in rural areas.

Our finding that the overall number of consultations is balanced between males and females is a positive one, potentially indicating that access to outpatient mental health services is equitable by gender. Specific mental disorders may have disparate population burdens by gender, so any claims around equity of access must take a disorder-specific and population-level approach. For example, females may have disproportionately lower access to treatment for substance use disorders given previous population-level studies estimating a 2:1 male to female ratio for current drinking [23], yet we found a 3.5:1 ratio for outpatient diagnoses of substance use disorders. A similar gender difference in access, utilization, or diagnosis patterns may exist for neurotic disorders given we found a 2.1:1 female to male ratio in diagnoses. Clear next steps are to estimate population burden of specific conditions in rural and urban Mozambique to target future interventions to ensure equitable mental healthcare access.

Mood and neurotic disorders represent a very low (and not increasing) proportion of consultations, which is potentially worrisome as these conditions are hypothesized to have the highest population prevalence and disability burden in Mozambique, based on modeling studies [22]. It may be that, similar to other LMIC settings, individuals are more likely to seek care from community supports and healers rather than allopathic care for common, less severe forms of mental illness [24]. While we do not advocate the replacement of strong community supports for individuals suffering from depression, anxiety, or other common mental disorders, there are likely many individuals who could benefit from brief interventions, psychotherapy, or psychotropic treatment who are currently not identified and served by the care system. Given the setting, the implementation of easy-to-use and rapid screening tools such as the Patient Health Questionnaire-2 for depression, which is reliable in diverse primary care settings [25], could be considered. Increased identification and treatment of depression may be particularly important in Mozambique as the WHO recently estimated Mozambique to have the highest suicide rate in Africa, and the 7th highest rate worldwide [6]. While no studies exist on the proportion of suicidal behavior in Mozambique attributable to major depressive disorder or other ICD-10-diagnosable conditions, it is likely that the expansion of formal treatment systems for these conditions could help prevent suicide and other forms of self-harm.

Across the two-year time period, we found a concerning association between psychiatric technicians serving at a facility and a higher rate of diagnoses for schizophrenia or delusional disorders. As this association remains after controlling for rural/urban status and other important facility-level factors, psychiatric technicians may be in need of further situational analysis and possibly additional training and/or supervision by psychiatrists or other highly-trained specialists to ensure patients are receiving the best clinical care possible. Based on conversations with other highly-trained providers in Mozambique, many believe that schizophrenia or delirium may be currently used as a catch-all diagnosis for individuals presenting with severe non-specific symptoms such as hallucinations, psychosis, delusions, or disorganized and/or aggressive behavior. A similar association between psychologists and lower rates of schizophrenia diagnoses, but higher rates of epileptic diagnoses, remains to be well understood.

While the majority of the increase in the absolute number of consultations over the last two years has been for epilepsy, there has been a positive trend of an increased diversity of diagnoses across the ICD-10 spectrum. This increased diversity of diagnoses has paralleled a large decrease in the absolute and proportionate amount of patients seen for schizophrenia or delusional disorders. We posit that across the follow-up period, as training levels, experience, and re-training of providers have progressed, it appears as though this may have translated into more specific, and thus, more accurate and effective diagnoses. However, it is also possible that technicians and clinics operating in rural areas are missing important cases of schizophrenia, and that the observed decrease in the number of diagnoses of schizophrenia reflects a decrease in population coverage for schizophrenia. Further studies are needed triangulating clinic and population-level data, along with gold-standard diagnostic comparators to allow disentangling changes in the case mix of patients from diagnostic skills and practices of mental health professionals.

When interpreting findings of this study it is important to keep in mind four major limitations. First, our analyses are based on aggregate estimates within ICD-10 categories, not individual patients, and thus there is potential for confounding or cross-level bias in our estimates. Second, the routine data system for mental health has not undergone any formal data quality audit procedures, as compared to other routine indicators in Mozambique [26], and therefore we cannot attest to the reliability or overall quality of these data. Although, a concurrent primary health care intervention [27] across the province has shown impressive and sustained increases in data quality for other routine health service indicators, with likely spill-over effects into other routine indicators [28]. Third, due to reporting issues, we are missing facility-level data for Beira City clinics, thus decreasing our sample size and potentially biasing results if these clinics are systematically different from those included in the sample. Fourth, as previously mentioned, we cannot discern if the observed changes in diagnostic patterns are due to shifts in case mix or changes in diagnostic skills or practices of clinicians. Last, given the small number of clusters (13 clinics) included in our time-series analyses and the small number of consultations for some ICD-10 categories, there was instability in some modelled estimates which necessitated exclusion of some ICD-10 codes from time-series analyses and staffing variables from most ICD-10 diagnostic categories.

This study also has a number of notable strengths. Namely, these data can be considered a census of mental health outpatient consultations conducted across public-sector clinics operating in 12 of 13 districts across a province of approximately two million people [29]. Also, since these data are collected through routine data systems rather than a survey, they may be less prone to the Hawthorne effect or other reactive biases and are continuous over time [30].

## Conclusion

Outpatient mental healthcare is rapidly expanding in Mozambique, yet still remains underfunded and under-resourced relative to the estimated population burden of mental disorders nationally. Utilization is increasing, especially in rural areas, but currently mostly serves individuals with epilepsy and schizophrenia/delusional disorders. Mood disorders, hypothesized to have a large population prevalence, are currently not well-addressed by the care system. Task-sharing approaches utilizing psychiatric technicians have allowed rapid expansion of the care system, although care provided by this cadre should be reviewed to ensure evidence-based diagnostic and treatment guidelines are being followed. Population-level surveys on the prevalence and care-seeking of common mental disorders are urgently needed to triangulate systems data with population burden to inform future efforts to expand equitable access to high-quality mental healthcare in Mozambique. Models and decision-support tools for mental healthcare integration with primary care practice are needed in Mozambique to allow further scale-up of mental health services.

## References

[1] Murray C, Lopez A, Jamison D (1994). The global burden of disease in 1990: summary results, sensitivity analysis, and future directions. Bull World Health Organ.

[2] World Health Organization (2001). The World Health Report 2001: Mental Health - New Understanding, New Hope.

[3] Prince M, Patel V, Saxena S, Maj M, Maselko J, Phillips MR (2007). No health without mental health. Lancet.

[4] World Health Organization (2011). Mental Health Atlas 2011.

[5] Saxena S, Thornicroft G, Knapp M, Whiteford H (2007). Resources for mental health: scarcity, inequity, and inefficiency. Lancet.

[6] World Health Organization (2014). Preventing Suicide - A Global Imperative.

[7] Dos Santos PF (2011). Avaliacao Dos Servicos de Saude Mental Em Mocambique.

[8] Department of Mental Health and Substance Abuse (2011). World Health Organization: *Mental Health Atlas 2011: Mozambique*.

[9] Patel V, Goel DS, Desai R (2009). Scaling up services for mental and neurological disorders in low-resource settings. Int Health.

[10] Rajaraman D, Travasso S, Chatterjee A, Bhat B, Andrew G, Parab S (2012). The acceptability, feasibility and impact of a lay health counsellor delivered health promoting schools programme in India: a case study evaluation. BMC Health Serv Res.

[11] World Health Organization (2008). Task Shifting: Rational Redistribution of Tasks among Health Workforce Teams: Global Recommendations and Guidelines.

[12] Eaton J, McCay L, Semrau M, Chatterjee S, Baingana F, Araya R (2011). Scale up of services for mental health in low-income and middle-income countries. Lancet.

[13] Patel V, Weiss HA, Chowdhary N, Naik S, Pednekar S, Chatterjee S (2010). Effectiveness of an intervention led by lay health counsellors for depressive and anxiety disorders in primary care in Goa, India (MANAS): a cluster randomised controlled trial. Lancet.

[14] Balaji M, Chatterjee S, Koschorke M, Rangaswamy T, Chavan A, Dabholkar H (2012). The development of a lay health worker delivered collaborative community based intervention for people with schizophrenia in India. BMC Health Serv Res.

[15] Peterson I, Bhana A, Baillie K (2012). The feasibility of adapted group-based interpersonal therapy (IPT) for the treatment of depression by community health workers within the context of task shifting in South Africa. Community Ment Health J.

[16] Van Ginneken N, Tharayan P, Lwein S, Rao G, Meera S, Pian J (2013). Non-specialist health worker interventions for the care of mental, neurological and substance-abuse disorders in low- and middle-income countries. Cochrane Database Syst Rev.

[17] World Health Organization (2010). mhGAP Intervention Guide for Mental, Neurological, and Substance Use Disorders in Non-Specialized Heath Settings.

[18] Mayeya J, Chazulwa R, Mayeya PN, Mbewe E, Magolo LM, Kasisi F (2004). Zambia mental health country profile. Int Rev Psychiatry.

[19] World Health Organization (1992). The ICD-10 Classification of Mental and Behavioral Disorders: Clinical Descriptions and Diagnostic Guidelines.

[20] Ustiin TB, Sartorius N: Results from the Ibadan Centre. In *Mental Illness in General Health Care: An International Study*; 1995:157–173.

[21] MISAU (2013). Relatorio Anual de Actividades de 2012.

[22] Institute for Health Metrics and Evaluation (IHME) (2013). GBD Compare Data Visualization: Mozambique.

[23] Padrão P, Damasceno A, Silva-Matos C, Laszczyńska O, Prista A, Gouveia L (2011). Alcohol consumption in Mozambique: regular consumption, weekly pattern and binge drinking. Drug Alcohol Depend.

[24] Wagenaar BH, Kohrt BA, Hagaman AK, McLean KE, Kaiser BN (2013). Determinants of care seeking for mental health problems in rural Haiti: culture, cost, or competency. Psychiatr Serv.

[25] World Health Organization (2009). Pharmacological Treatment of Mental Disorders in Primary Health Care.

[26] Gimbel S, Micek M, Lambdin B, Lara J, Karagianis M, Cuembelo F (2011). An assessment of routine primary care health information system data quality in Sofala Province, Mozambique. Popul Health Metr.

[27] Sherr K, Cuembelo F, Michel C, Gimbel S, Micek M, Kariaganis M (2013). Strengthening integrated primary health care in Sofala, Mozambique. BMC Health Serv Res.

[28] Wagenaar BH, Gimbel S, Hoek R, Pfeiffer J, Michel C, Manuel JL (2015). Effects of a health information system data quality intervention on concordance in Mozambique: time-series analyses from 2009–2012. Popul Health Metr.

[29] National Statistics Institute of Mozambique (2007). Mozambique Census.

[30] Wagenaar BH, Sherr K, Fernandes Q, Wagenaar AC: Using routine health information systems for well-designed health evaluations in low- and middle-income countries. *Health Policy Plan* 2015:1–7. doi:10.1093/heapol/czv029 10.1093/heapol/czv029 PMC4751224 25887561

